# Combined Anterior Cruciate Ligament and Anterolateral Ligament Reconstruction Shows Reduced Graft Failure Rates and Superior Residual Rotational Stability Regardless of Anterolateral Ligament Reconstruction Graft: A Systematic Review

**DOI:** 10.3390/jcm14072237

**Published:** 2025-03-25

**Authors:** Joo Hyung Han, Sung-Hwan Kim, Min Jung, Hyun-Soo Moon, Kwangho Chung

**Affiliations:** 1 Yonsei University College of Medicine, Seoul 03722, Republic of Korea; hanjh93@gmail.com; 2 Arthroscopy and Joint Research Institute, Yonsei University College of Medicine, Seoul 03722, Republic of Korea; orthohwan@gmail.com (S.-H.K.); oshsdesu@gmail.com (H.-S.M.); 3 Department of Orthopedic Surgery, Gangnam Severance Hospital, Yonsei University College of Medicine, Seoul 03722, Republic of Korea; 4 Department of Orthopedic Surgery, Severance Hospital, Yonsei University College of Medicine, Seoul 03722, Republic of Korea; 5 Department of Orthopedic Surgery, Yongin Severance Hospital, Yonsei University College of Medicine, Yongin 16995, Republic of Korea

**Keywords:** anterior cruciate ligament reconstruction, anterolateral ligament reconstruction, graft failure, residual rotational stability, systematic review

## Abstract

**Objectives**: The aim of this study is to evaluate the literature for comparing clinical outcomes of anterior cruciate ligament reconstruction (ACLR) with concomitant anterolateral ligament reconstruction (ALLR) versus isolated ACLR, with a primary focus on analyzing differences in outcomes based on the type of graft used for ALLR. **Methods**: We identified comparative studies involving primary ACLR performed in conjunction with ALLR. Graft failure rates, residual pivot shift, residual anterior–posterior (AP) laxity at follow-up, and patient-reported outcome measures were determined. Variables associated with isolated ACLR and ACLR combined with ALLR were compared based on the type of graft used for ALLR. **Results**: This systematic review included nine studies involving 2740 patients. Combined ACLR with ALLR using hamstring tendon (HT) autografts or tibialis allografts showed lower graft failure rates than isolated ACLR (HT autograft: rate, 0–5.9%, odds ratio [OR], 2.16–12.91; tibialis allograft: rate, 0%, OR, 2.00–5.27). Similarly, the combined procedure showed reduced residual pivot shift rates (HT autograft: rate, 0–9.1%, OR, 2.00–12.16; tibialis allograft: rate, 0%, OR, 7.65–15.33) compared to isolated ACLR. Residual AP laxity and patient-reported outcomes were similar or more favorable for the combined procedure; however, the results were heterogeneous. Complications related to the type of graft used for ALLR or the presence of ALLR itself were not reported. **Conclusions**: Regardless of the graft type used for ALLR, the combination of ACLR with ALLR showed better clinical outcomes, including reduced graft failure rates and superior residual rotational stability compared to isolated ACLR. However, the high heterogeneity observed across studies suggests that these findings should be interpreted with caution, and further research is needed to draw more definitive conclusions.

## 1. Introduction

Anterior cruciate ligament (ACL) rupture, one of the most frequently injured ligaments in the knee joint, is effectively treated using ACL reconstruction (ACLR) [1,2]. However, graft failure requiring revision surgery remains an unresolved issue. It is reported in 4–25% of patients who have undergone ACLR, with the majority of cases noted within two years after surgery [3,4,5,6,7]. Persistent rotational instability following ACLR is recognized not only as a frequent cause of graft failure but also as a factor that adversely affects patient outcomes [8].

Anterolateral ligament (ALL) reconstruction (ALLR), in conjunction with ACLR, has been proposed to address residual rotational instability that cannot be controlled by ACLR alone. The biomechanics and functions of the ALL have been investigated in several studies. The results indicate that the ALL acts as a secondary stabilizer to the ACL, especially in resisting anterior tibial translation and internal rotation, thus playing a vital role in preserving rotational stability and preventing knee instability [9,10]. Compared with isolated ACLR, combined ACLR and ALLR enhances rotational stability, assessed through the pivot shift test [9]. Furthermore, the combination reduces graft rates and enhances subjective function scores [11].

Graft selection for ACL is one of the most extensively discussed topics to date, with substantial evidence supporting various graft options, leading to different outcomes depending on the type of graft utilized [12,13,14]. When comparing autografts and allografts for ACLR, autografts, such as hamstring grafts and bone–patellar tendon–bone (BPTB) grafts, are generally considered superior to allografts in terms of graft failure rates, which can be up to six times higher with allografts, particularly in younger more active patients [15,16,17,18].

Several systematic reviews and meta-analyses have consistently shown that combining ACLR with anterolateral ligament reconstruction (ALLR) results in improved outcomes compared to isolated ACLR [11,19,20]. While the integration of ALLR into ACLR has become increasingly prevalent, with various graft types being utilized for ALLR [12], there remains a lack of evidence and literature to support specific graft choices and their impact on clinical outcomes.

This systematic review sought to assess the available literature for a comparison of the clinical outcomes of combined ACLR and ALLR versus isolated ACLR, with a primary focus on assessing differences in outcomes based on the type of graft used for ALLR. We hypothesize that ACLR combined with ALLR will consistently yield superior clinical outcomes compared to isolated ACLR, regardless of the graft type used for ALLR.

## 2. Materials and Methods

### 2.1. Search Strategy

The review was registered in advance in the PROSPERO prospective register of systematic reviews (ID: CRD42023482821) and was carried out according to a predefined protocol following the PRISMA (Preferred Reporting Items for Systematic Reviews and Meta-analyses) guidelines. A thorough search strategy was developed to identify relevant studies. We conducted a systematic search of PubMed, Embase, the Cochrane Library, and Google Scholar for articles published up to 11 September 2024. The search was conducted utilizing the following search parameters: [(“antero lateral ligament” OR “anterolateral ligament” OR “ALL”) AND (“anterior cruciate ligament” [mesh] OR “anterior cruciate ligament” OR “ACL”) AND “reconstruction”]. The search was restricted to studies published in the English language.

### 2.2. Identification of Eligibility

The inclusion criteria were as follows: (1) skeletally mature patients aged 15 to 50 years diagnosed with ACL rupture; (2) studies involving the intervention of primary single-bundle ACLR with ALLR; (3) comparative studies with levels of evidence I to III, published in English; (4) studies with a minimum follow-up period of 24 months; and (5) studies specifying the graft type used for ALLR. The exclusion criteria were as follows: (1) articles not in English; (2) studies with incomplete data; (3) studies involving revision ACLR; and (4) studies including lateral extra-articular tenodesis; and (5) level IV to V studies, including cadaveric, animal studies, case reports, systematic reviews, or biomechanical studies. Eligibility was determined by two independent reviewers who screened the search results.

### 2.3. Data Extraction

Two independent reviewers extracted data including the following factors: primary author, publication year, country of origin, study design, level of evidence, type of graft used for ALLR, sample size, duration of follow-up, sex distribution, age, body mass index, reported ACLR graft failure rate, residual pivot shift at follow-up, residual anterior–posterior (AP) laxity, and patient-reported outcome measures, such as pre-and postoperative International Knee Documentation Committee (IKDC) scores, Lysholm scores, and Tegner scores, along with documented complications. In the case of disagreement between the reviewers, discrepancies were resolved via discussion until a consensus was reached. If no agreement could be reached, a third reviewer was consulted.

To assess the risk of bias, randomized controlled trials (RCTs) were evaluated using the Cochrane Collaboration’s Risk of Bias 2 tool [21]. Each study was categorized with a low, unclear, or high risk of bias. The risk of bias for non-randomized studies was assessed using the Methodological Index for Non-Randomized Studies (MINORS), which includes 12 criteria for comparative studies [22]. Each category was rated as 0 (if not reported), 1 (if reported but inadequate), or 2 (if reported and considered adequate). The risk of bias was independently assessed by two reviewers, with disagreements resolved via consultation with a third reviewer.

### 2.4. Statistical Analysis

Descriptive statistics, including the mean and standard deviation for numerical variables, were recorded. For studies that did not provide the standard deviation, we calculated it using other available statistical values, following the method outlined by Furukawa et al. [23].

In cases where results were presented only as graphs without numerical values, we employed the approach described by Gheibi et al. to derive numerical data for analysis [24]. For each investigated variable, we calculated the differences in clinical efficacy between ACLR with concomitant ALLR and isolated ACLR. Residual AP laxity was represented by the side-to-side difference in millimeters as reported in each study, while residual pivot shift was defined as cases exhibiting a grade 2 or 3 pivot shift. For continuous outcome measures, mean differences (MDs) with 95% confidence intervals (CIs) were utilized. Odds ratios (ORs) were calculated for dichotomous outcomes, with 95% CIs. Studies from the same research group, where there was a potential overlap of patients in the intervention groups, were excluded from the analysis, with the exception of the study with the largest sample size. Heterogeneity between studies was assessed using the Q statistic and the I^2^ statistic. While a meta-analysis was considered, its feasibility was limited by the relatively low level of evidence and high heterogeneity across studies. As a result, pooled synthesis was avoided. Instead, Forest plots were generated for outcomes including graft failure rate, residual AP laxity, residual pivot shift, and patient-reported outcome measures, with subgroup analysis based on the type of graft utilized in ALLR. All statistical analyses and data visualizations were performed using R software (version 4.2.1; R Foundation, Vienna, Austria).

## 3. Results

### 3.1. Characteristics of Included Studies

**Figure 1:**
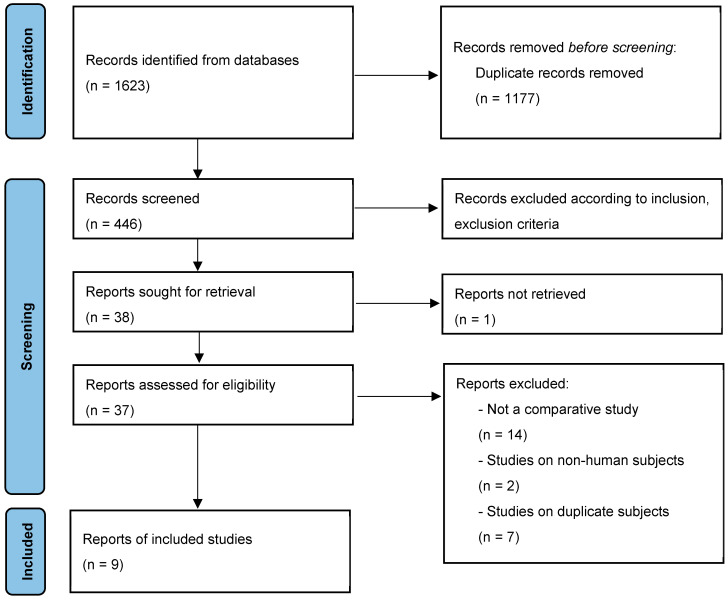
PRISMA flow diagram for the systematic review.

This systematic review identified 1623 relevant studies from multiple databases. After eliminating duplicates and assessing the full texts, 37 studies were considered for eligibility. Ultimately, nine studies, involving 2740 patients in total, fulfilled our inclusion criteria and were included in the analysis (Figure 1) [25,26,27,28,29,30,31,32,33]. The characteristics of these included studies are detailed in Table 1 and Table 2. Based on the graft utilized in ALLR, seven studies [25,27,28,29,30,31,32] used a hamstring tendon (HT) autograft, and two studies [26,33] employed a tibialis allograft.

### 3.2. Methodological Quality Assessment of Included Studies

**Figure A1:**
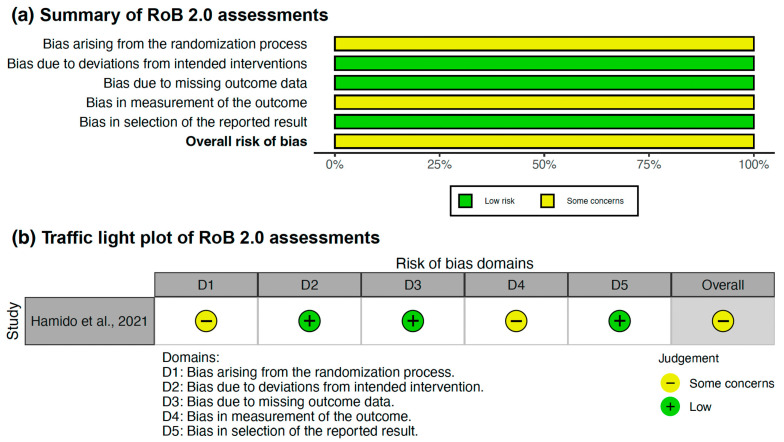
Summary of the risk of bias assessment for included studies. (**a**) Summary of the RoB 2.0 assessment. (**b**) Traffic light plot for the RoB 2.0 assessment. RoB, risk of bias [30].

Among the included studies, one study [30] was classified as level I evidence, two studies [31,33] as level II evidence, and six studies [25,26,27,28,29,32] as level III evidence. One study [30], classified as an RCT, was evaluated using the Cochrane Collaboration’s Risk of Bias 2 tool, and the results are presented in Figure A1. The remaining eight studies [25,26,27,28,29,31,32,33] were assessed using the MINORS tool, with a mean score of 21.4 ± 1.5 out of a maximum of 24 points, indicating generally high methodological quality. Further details on the MINORS scores are provided in Table A1. The inclusion criteria used in each study are summarized in Table A2.

### 3.3. Graft Failure

**Figure 2:**
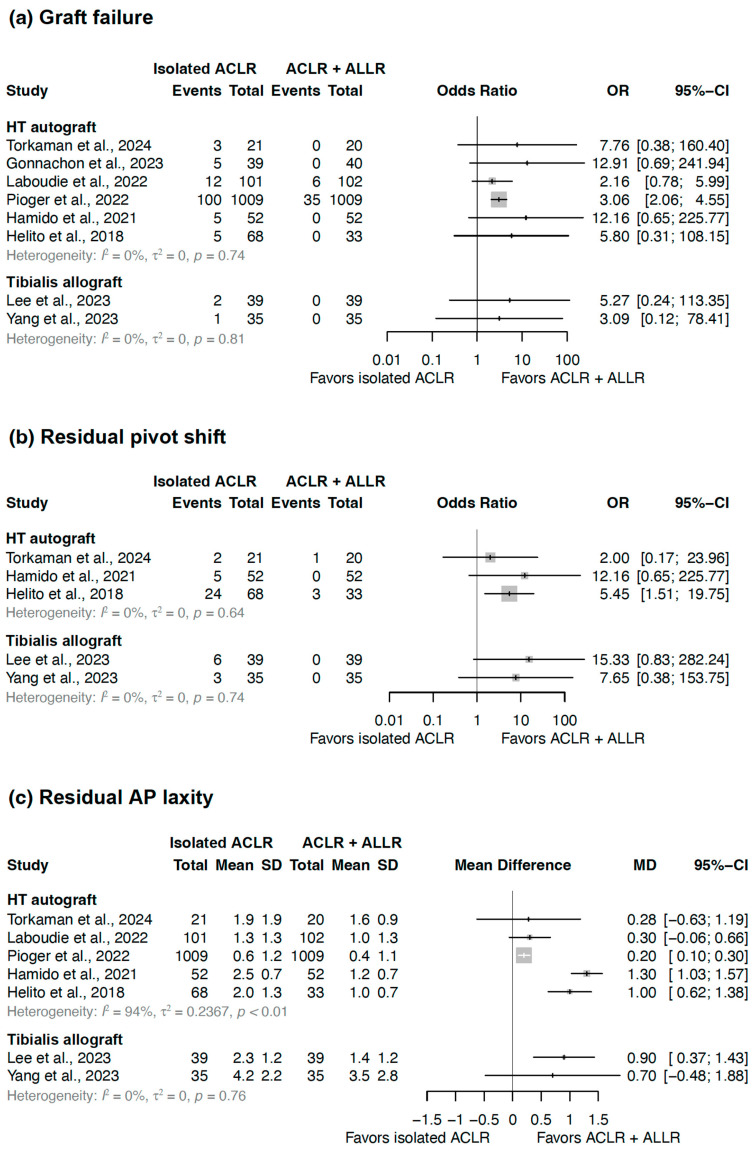
Forest plot illustrating the differences in treatment effects between isolated ACLR and cases in which ACLR and ALLR were performed concurrently, divided into subgroups according to the ALLR graft used. The results for (**a**) the failure rate of the ACLR graft, (**b**) residual pivot shift, and (**c**) residual anterior–posterior (AP) laxity are presented. Gray squares indicate the effect estimates for each study, with their size representing the study's weight in the analysis. ACLR, anterior cruciate ligament reconstruction; ALLR, anterolateral ligament reconstruction; SD, standard deviation; MD, mean difference; CI, confidence interval; HT, hamstring tendon [25,26,27,28,29,30,32,33].

Graft failure rates have been reported in eight studies (Table 3) [25,26,27,28,29,30,32,33]. Combined ACLR with ALLR had a lower graft failure rate (0–5.9%) compared to isolated ACLR (2.9–14.3%), with an OR ranging from 2.16 to 12.91. The comparison of combined ACLR with ALLR to isolated ACLR based on the graft used for ALLR showed that HT autograft [27,28,29,30,32,33] had an OR of 2.16–12.91, whereas tibialis allograft [25,26] had an OR of 2.00–5.27 (Figure 2a). Both HT autograft (I^2^ = 0%, τ^2^ = 0.0, *p* = 0.74) and tibialis allograft (I^2^ = 0%, τ^2^ = 0.0, *p* = 0.81) exhibited low heterogeneity.

### 3.4. Residual Pivot Shift

Residual pivot shift rates have been reported in five studies (Table 3) [25,26,30,32,33]. Combined ACLR with ALLR had a lower residual pivot shift rate (0–9.1%) compared to isolated ACLR (8.6–35.3%), with an OR ranging from 2.00 to 15.33. The comparison of combined ACLR with ALLR to isolated ACLR based on the graft used for ALLR showed that HT autograft [30,32,33] had an OR of 2.00–12.16, whereas tibialis allograft [25,26] had an OR of 7.65–15.33 (Figure 2b). Both HT autograft (I^2^ = 0%, τ^2^ = 0.0, *p* = 0.64) and tibialis allograft (I^2^ = 0%, τ^2^ = 0.0, *p* = 0.74) exhibited low heterogeneity.

### 3.5. Residual Anterior–Posterior Laxity

Residual AP laxity has been reported in seven studies (Table 3) [25,26,28,29,30,32,33]. The comparison of combined ACLR with ALLR with isolated ACLR based on the graft used for ALLR showed that for HT autografts [28,29,30,32,33], MDs ranged from 0.20 to 1.30, whereas for tibialis allografts [25,26], MDs ranged from 0.70 to 0.90 (Figure 2c). HT autografts (I^2^ = 94%, τ^2^ = 0.2367, *p* < 0.01) exhibited high heterogeneity, whereas tibialis allografts (I^2^ = 0%, τ^2^ = 0.0, *p* = 0.76) exhibited low heterogeneity.

### 3.6. Patient-Reported Outcome Measures

**Figure 3:**
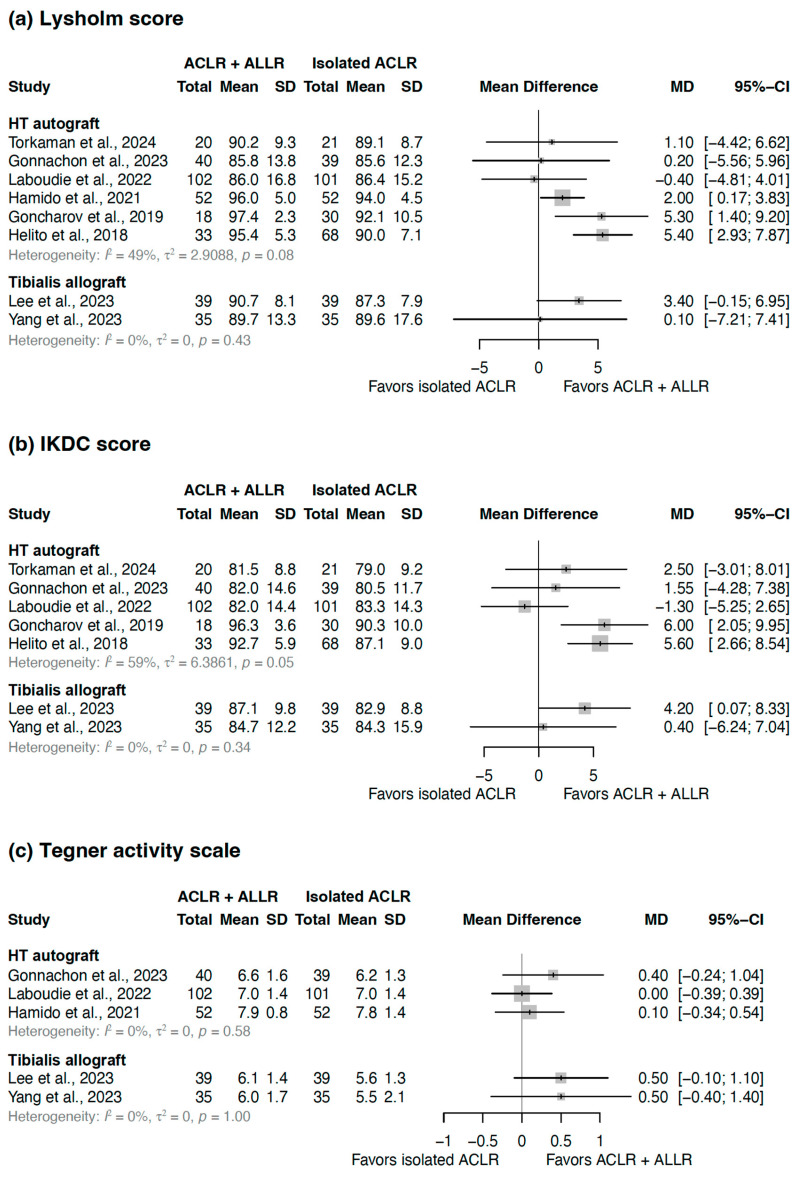
Forest plot illustrating the differences in treatment effects between isolated ACLR and cases in which ACLR and ALLR were performed concurrently, divided into subgroups according to the ALLR graft used. The results for (**a**) Lysholm score, (**b**) IKDC score, and (**c**) Tegner activity scale are presented. Gray squares indicate the effect estimates for each study, with their size representing the study's weight in the analysis. ACLR, anterior cruciate ligament reconstruction; ALLR, anterolateral ligament reconstruction; SD, standard deviation; MD, mean difference; CI, confidence interval; HT, hamstring tendon; IKDC, International Knee Documentation Committee [25,26,27,29,30,31,32,33].

Patient-reported outcome measures included the Lysholm and IKDC scores and the Tegner activity scale (Table 4). The Lysholm score has been reported in eight studies [25,26,27,29,30,31,32,33]. The comparison of combined ACLR with ALLR with isolated ACLR based on the graft used for ALLR showed that for HT autografts [27,29,30,31,32,33], MDs ranged from −0.40 to 5.40, whereas for tibialis allografts [25,26], MDs ranged from 0.10 to 3.40 (Figure 3a). HT autografts (I^2^ = 49%, τ^2^ = 2.9088, *p* = 0.08) exhibited moderate heterogeneity, whereas tibialis allografts (I^2^ = 0%, τ^2^ = 0.0, *p* = 0.43) exhibited low heterogeneity.

The IKDC score has been reported in seven studies [25,26,27,29,31,32,33]. The comparison of combined ACLR with ALLR with isolated ACLR based on the graft used for ALLR showed that for HT autografts [27,29,31,32,33], MDs ranged from −1.30 to 6.00, whereas for tibialis allografts [25,26], MDs ranged from 0.40 to 4.20 (Figure 3b). HT autografts (I^2^ = 59%, τ^2^ = 6.3861, *p* = 0.05) exhibited high heterogeneity, whereas tibialis allografts (I^2^ = 0%, τ^2^ = 0.0, *p* = 0.34) exhibited low heterogeneity.

The Tegner activity scale has been reported in five studies [25,26,29,30,31]. The comparison of combined ACLR with ALLR with isolated ACLR based on the graft used for ALLR showed that for HT autografts [27,29,30], MDs ranged from 0.00 to 0.40, whereas for tibialis allografts [25,26], the MD of 0.50 was reported (Figure 3c). Both HT autografts (I^2^ = 0%, τ^2^ = 0.0, *p* = 0.58) and tibialis allografts (I^2^ = 0%, τ^2^ = 0.0, *p* = 1.00) exhibited low heterogeneity.

### 3.7. Complications

Complications have been reported in seven studies [25,26,27,28,29,30,32]. For HT autografts for ALLR, Gonnachon et al. [27] and Hamido et al. [30] reported no major complications in either the isolated ACLR group or the combined ACLR and ALLR group. Laboudine et al. [29] observed no difference in occasional knee pain, hardware problems, stiffness, swelling, or cyclops lesion between the two groups two years after surgery. Pioger et al. [28] reported that reoperations for symptomatic cyclops lesions and secondary meniscectomy were more frequent in the isolated ACLR group. Helito et al. [32] reported that one patient in the isolated ACLR group had a hypertrophic scar on the thigh due to the outside-in technique, and one patient had a pretibial cyst. In the ACLR with ALLR group, one patient experienced femoral anchor loosening with irritation of the lateral soft tissue of the knee, requiring removal. Torkaman et al. [33] reported that one patient in the isolated ACLR group and one patient in the combined ACLR and ALLR group developed postoperative arthrofibrosis, but both regained full range of motion within six months. Additionally, eight patients in the isolated ACLR group and seven in the combined group experienced anterior knee pain, with no statistically significant difference in complications between the groups.

For studies using tibialis allografts for ALLR, Lee et al. [26] reported no sustained pain, bioabsorbable screw protrusion, stiffness, or swelling at a minimum two-year follow-up. Yang et al. [25] noted that in the ACLR with ALLR group, one patient had a cyclops-type lesion and one patient had a superficial wound infection. However, all patients in both groups recovered by the final follow-up without specific complications.

## 4. Discussion

The primary findings of this systematic review revealed that regardless of the graft type used for ALLR, combining ACLR with ALLR resulted in reduced graft failure rates and superior residual rotational stability compared to isolated ACLR. Additionally, residual AP stability and patient-reported outcome measures were generally favorable or comparable for the combined procedure; however, the results were heterogeneous across studies. Notably, no complications related to the specific graft type used for ALLR or the presence of ALLR were reported. Future research, particularly studies directly comparing graft types for ALLR, is needed to better define the influence of graft selection on the outcomes of combined ACLR and ALLR procedures.

The primary objective of introducing ALLR was to address persistent rotatory instability, a common issue associated with isolated ACLR, which ultimately aims to prevent ACL graft failure [34]. Several systematic reviews and meta-analyses have consistently reported that the combined ACLR and ALLR procedure is superior to isolated ACLR in terms of graft failure, residual pivot shift, and clinical outcomes [11,19,20]. Consistent with other reviews, our study also demonstrates that combined reconstruction is superior to isolated ACLR in terms of graft failure and residual pivot shift.

Graft selection, whether autografts or allografts, is recognized as one of the critical modifiable factors that influence surgical outcomes, such as graft failure, following ACLR [5]. In their randomized controlled trial comparing tibialis posterior allografts and hamstring autografts for ACLR, Bottoni et al. [15] demonstrated that the allograft had a failure rate over three times higher than the autograft at a minimum ten-year follow-up. Similarly, data from the Kaiser Permanente ACLR Registry indicated that BPTB allografts had an overall 4.5-fold higher risk of revision compared to BPTB autografts for ACLR [35]. However, these higher failure rates of allografts compared to those of autografts have not been consistent in some recent studies, possibly due to the use of fresh frozen allografts instead of irradiated allografts [36]. The ACL grafts used in the studies included in this review are predominately autologous hamstring grafts, with only one study using a BPTB graft. The impact of the included BPTB autograft is considered negligible, as there is no statistically significant difference in graft failure rates, even though HT grafts tend to exhibit a higher failure rate [37,38,39].

Given the impact of graft selection on ACLR outcomes, it is essential to examine how graft choice may similarly affect ALLR and, consequently, influence the overall outcomes of ACLR. Despite this, limited research exists regarding the optimal graft choice for ALLR, with few studies investigating this issue. Previous meta-analyses related to our study topic have explored ALLR, lateral extra-articular procedures, and their various clinical outcomes [11,19,20]. However, none have conducted a detailed analysis of differences based on ALLR graft selection. Therefore, this study aims to provide insights into this aspect, which has not been addressed in prior systematic reviews or meta-analyses. While various autograft candidates for ALLR, including the iliotibial band and the gracilis, semitendinosus, plantaris longus, quadriceps, and patellar tendons, have been proposed [40], the HT, predominantly the one-strand gracilis tendon, was the most commonly used autograft for ALLR performed concurrently with ACLR in this review [27,28,29,30,31,32,33].

In their cadaveric study, Wytrykowski et al. [41] compared the mechanical properties of ALL with potential graft candidates for ALLR, including the gracilis tendon and iliotibial band. They observed that the two-strand gracilis tendon exhibited the highest maximum load to failure at 201 N compared to the native ALL at 141 N and the iliotibial band at 161 N. Considering that a four-strand gracilis tendon has a maximum load to failure of 416 N, which is double that of a two-strand gracilis, it can be inferred that a one-strand gracilis would have half or less than half of the maximum load that a two-strand gracilis construct can withstand [42]. Consequently, the one-strand gracilis tendon used in most of the included studies may not attain the strength of the native ALL, as it is estimated to have a maximum load of approximately 100 N. This insufficient graft strength may limit its ability to recapitulate the role of the ALL, potentially leading to no significant difference in graft failure or residual pivot shift between autografts and allografts in the current review; however, it may still yield superior outcomes than isolated ACLR. Additionally, while it is difficult to determine the exact properties of the grafts used in each study included in this review, reports suggest that tibialis allografts can serve as substitutes for hamstring tendons based on their mechanical properties [43]. This supports the finding that there was no clear difference between autografts and allografts in the review.

Allografts can be a viable graft option for ALLR, particularly when autograft options have been exhausted in multi-ligament or revision reconstructions or when performing both ACLR and ALLR with a hamstring autograft results in suboptimal ACL or ALL graft characteristics [40]. In cases of combined ACLR and ALLR using a hamstring autograft, as in the studies included in our review, most grafts, such as a three-strand semitendinosus tendon and one-strand gracilis tendon or a three- or four-strand semitendinosus tendon, are used for ACLR, leaving only a one- or two-strand gracilis tendon for ALLR [25,26,27,28,29,30,31,32,33]. These graft constructs present a potential risk of a small ACL graft diameter, even with a three- or four-strand ACL graft. In an attempt to compensate by creating a three- or four-strand construct, the ACL graft tunnel length may become shortened, often leaving only a one-strand gracilis tendon available for use as the ALL graft [44]. The ACL graft diameter has considerable implications for graft failure, as every 0.5 to 1.0 mm increase in diameter is associated with an approximately 20% decrease in the graft failure rate [45,46]. Furthermore, short graft tunnel length raises concerns regarding graft fixation, stability, and the risk of tunnel enlargement [47,48]. Given that our study revealed no significant differences in graft rupture rates, rotational stability, or patient-reported outcomes between autografts and allografts, using an allograft for ALLR may be particularly useful for surgeons who wish to utilize hamstring autograft for ACLR without compromising graft characteristics.

Common issues associated with the use of allografts include concerns regarding disease transmission and increased cost [49]. However, the studies included in this review did not report complications specific to allografts or ALLR. This absence of complications could be attributed to the rarity of disease transmission today due to more stringent donor screening and testing protocols [49]. Furthermore, unlike lateral extra-articular tenodesis, ALLR demonstrates a lower risk of overconstraint-related issues, such as knee stiffness, which may account for the lack of reported complications [11]. However, the increased cost associated with its use should be thoroughly discussed with the patient prior to decision-making. Our findings may serve to guide surgeons in selecting the most appropriate graft type based on the specific clinical scenario and the unique characteristics of each graft, thereby potentially optimizing patient outcomes.

The residual AP laxity reported in several studies included in this review generally demonstrated either more favorable or comparable results for combined ACLR and ALLR compared to those for isolated ACLR. This finding aligns with previous research suggesting that the ALL works synergistically with the ACL to maintain both rotatory and AP stability of the knee [50]. However, in this review, a subgroup analysis based on the type of ALLR graft utilized revealed that HT autografts (I^2^ = 94%, τ^2^ = 0.2367, *p* < 0.01) exhibited high heterogeneity. Consequently, there is insufficient evidence to draw definitive conclusions regarding AP laxity differences related to ALLR graft types.

Patient-reported outcome measures, including the Lysholm and IKDC scores, and the Tegner activity scale demonstrated similar or higher outcome scores when both ACLR and ALLR were performed compared to isolated ACLR. However, for HT autografts, the Lysholm score showed moderate heterogeneity (I^2^ = 49%, τ^2^ = 2.9088, *p* = 0.08) and the IKDC score exhibited high heterogeneity (I^2^ = 59%, τ^2^ = 6.3861, *p* = 0.05).

The observed heterogeneity across studies may stem from differences in surgical techniques, graft preparation and fixation methods, rehabilitation protocols, and indications for ALLR. Variations in patient demographics, follow-up durations, and outcome assessment methods also likely contributed to inconsistencies. Additionally, the risk of bias for each study was assessed, but it is important to note that there is only one study with a level-I-evidence RCT, and differences in the inclusion criteria used across studies may also contribute to potential sources of bias. Given these factors, the results should be interpreted with caution.

### Limitations

Our study has some limitations. First, a few high-level evidence studies are available in this domain, particularly those focusing on the outcomes of using allografts in ALLR, which prevents the possibility of conducting a pooled synthesis. To address this, we presented the results by dividing the grafts into subgroups and providing a range of effect sizes through a forest plot. Second, only ALLR performed with primary ACLR was included, excluding studies involving other lateral extra-articular procedures or revision cases. Different lateral procedures may introduce heterogeneity due to anatomical differences from ALLR, and revision cases could represent a distinct clinical entity, potentially affecting outcomes such as graft failure. Therefore, our inclusion criteria were deemed appropriate for evaluating the specific function of the ALLR graft. Third, indications for ALLR differ across studies, which may have introduced heterogeneity in the analysis. Fourth, study design limitations made it impossible to directly compare the outcomes of autografts and allografts. Fifth, surgical outcomes can also be influenced by patient-specific factors such as age, activity level, and concomitant procedures, which necessitates caution when interpreting the results. Sixth, many studies in the analysis had relatively short- to medium-term follow-up periods, underscoring the need for future studies assessing long-term outcomes. Lastly, the number of studies using allografts as the ALLR graft was relatively small. Future studies should focus on directly comparing autografts and allografts or conducting a systematic review that includes more studies to enable more definitive conclusions.

## 5. Conclusions

Regardless of the graft type used for ALLR, the combination of ACLR with ALLR showed better clinical outcomes, including reduced graft failure rates and superior residual rotational stability compared to isolated ACLR. However, the high heterogeneity observed across studies suggests that these findings should be interpreted with caution, and further research is needed to draw more definitive conclusions.

## Figures and Tables

**Table 1:** Overview of included studies.

Study	Year	Journal	Country	Study Design	Level of Evidence	Number of Patients	Graft Used for ACLR	Graft Used for ALLR
Torkaman et al. [33]	2024	*BMC Musculoskeletal. Disord.*	Iran	Prospective comparative study	2	41	4 strand ST	1 strand GT
Gonnachon et al. [27]	2023	*Eur. J. Orthop. Surg. Traumatol.*	France	Cohort study	3	79	3 or 4 strand ST + GT	1 strand ST
Lee et al. [26]	2023	*Orthop. J. Sports Med.*	Republic of Korea	Cohort study	3	78	4 strand ST	TA allograft
Yang et al. [25]	2023	*Arthroscopy*	Republic of Korea	Cohort study	3	70	4 strand ST + GT	Tibialis allograft
Laboudie et al. [29]	2022	*Knee Surg. Sports Traumatol. Arthrosc.*	France	Retrospective comparative study	3	203	4 strand ST	1 strand GT
Pioger et al. [28]	2022	*Am. J. Sports Med.*	France	Cohort study	3	2018	4 strand ST + GT	1 strand GT
Hamido et al. [30]	2021	*Knee Surg. Sports Traumatol. Arthrosc.*	Kuwait	Randomized controlled trial	1	102	4 strand ST	2 strand GT
Goncharov et al. [31]	2019	*Int. Orthop.*	Russia	Prospective comparative study	2	48	BPTB Autograft	ST + GT
Helito et al. [32]	2018	*Knee Surg. Sports Traumatol. Arthrosc.*	Brazil	Cohort study	3	101	4 strand ST + GT	1 strand GT

ACLR, anterior cruciate ligament reconstruction; ALLR, anterolateral ligament reconstruction; ST, semitendinosus tendon; GT, gracilis tendon; BPTB, bone patellar tendon bone; TA, tibialis anterior.

**Table 2:** Demographics of patients in included studies.

Graft Used for ALLR	Study	Follow-Up Duration (Mean ± SD)	Sex (n, Male/Female)	Age (Years, Mean ± SD)	BMI (kg/m^2^, Mean ± SD)
HT Autograft	Torkaman et al., 2024 [33]	39.8 ± 14.1 (isolated ACLR), 41.3 ± 15 (ACLR + ALLR) months	17/4 (isolated ACLR), 18/2 (ACLR + ALLR)	26.7 ± 8.9 (isolated ACLR), 25.9 ± 6.9 (ACLR + ALLR)	24.5 ± 2.2 (isolated ACLR), 24.2 ± 2.1 (ACLR + ALLR)
	Gonnachon et al., 2023 [27]	54 months	67/16	24 ± 7.2	NR
	Laboudie et al., 2022 [29]	4.8 ± 0.9 years	57/44 (isolated ACLR), 62/40 (ACLR + ALLR)	16.5 ± 2.2 (isolated ACLR), 16.8 ± 1.9 (ACLR + ALLR)	21.8 ± 3 (isolated ACLR), 22.1 ± 3 (ACLR + ALLR)
	Pioger et al., 2022 [28]	101.3 ± 59.9 months	840/169 (isolated ACLR), 845/163 (ACLR + ALLR)	25.8 ± 7.5 (isolated ACLR), 25.8 ± 7.9 (ACLR + ALLR)	24.4 ± 3.4 (isolated ACLR), 24.3 ± 3.4 (ACLR + ALLR)
	Hamido et al., 2021 [30]	median 60 months (range 55–65)	52/0 (isolated ACLR), 50/0 (ACLR + ALLR)	26 (isolated ACLR), 24 (ACLR + ALLR)	NR
	Goncharov et al., 2019 [31]	24 months	NR	NR	NR
	Helito et al., 2018 [32]	26 (isolated ACLR), 25 (ACLR + ALLR) months	59/9 (isolated ACLR), 30/3 (ACLR + ALLR)	33.9 ± 6.1 (isolated ACLR), 33.1 ± 8.8 (ACLR + ALLR)	NR
Tibialis Allograft	Lee et al., 2023 [26]	30.4 ± 3.9 (isolated ACLR), 29.3 ± 3.5 (ACLR + ALLR) months	0/39 (isolated ACLR), 0/39 (ACLR + ALLR)	31.1 ± 5.7 (isolated ACLR), 30.4 ± 6.1 (ACLR + ALLR)	19.4 ± 2.5 (isolated ACLR), 19.7 ± 2.7 (ACLR + ALLR)
	Yang et al., 2023 [25]	46.6 months, 42.5 months	31/4 (isolated ACLR), 29/6 (ACLR + ALLR)	26.8 ± 9.9 (isolated ACLR), 26.1 ± 10.8 (ACLR + ALLR)	26.2 ± 4.6 (isolated ACLR), 26.6 ± 4.5 (ACLR + ALLR)

ALLR, anterolateral ligament reconstruction; SD, standard deviation; BMI, body mass index; HT, hamstring tendon, ACLR, anterior cruciate ligament reconstruction; NR, not reported.

**Table 3:** Graft failure rate, residual pivot shift rate, and residual AP laxity reported in included studies.

Graft Used for ALLR	Study	Graft Failure		Residual Pivot Shift		Residual AP Laxity (mm, Mean ± SD)		
		Isolated ACLR	ACLR + ALLR	Isolated ACLR	ACLR + ALLR	Isolated ACLR	ACLR + ALLR	Method
HT Autograft	Torkaman et al., 2024 [33]	3/21 (14.3%)	0/20 (0%)	2/21 (9.5%)	1/20 (5.0%)	1.9 ± 1.9	1.7 ± 0.9	KT-1000
	Gonnachon et al., 2023 [27]	5/39 (12.8%)	0/40 (0%)	NR	NR	NR	NR	NR
	Laboudie et al., 2022 [29]	12/101 (11.9%)	6/102 (5.9%)	NR	NR	1.3 ± 1.3	1 ± 1.3	GNRB
	Pioger et al., 2022 [28]	100/1009 (9.9%)	35/1009 (3.5%)	NR	NR	0.6 ± 1.2	0.4 ± 1.1	Rolimeter
	Hamido et al., 2021 [30]	5/52 (9.6%)	0/50 (0%)	5/52 (9.6%)	0/50 (0%)	2.5 ± 0.7	1.2 ± 0.7	KT-1000
	Goncharov et al., 2019 [31]	NR	NR	NR	NR	NR	NR	NR
	Helito et al., 2018 [32]	5/68 (7.4%)	0/33 (0%)	24/68 (35.3%)	3/33 (9.1%)	2 (95% CI 1.5–2.1)	1 (95% CI 1.14–1.6)	KT-1000
Tibialis Allograft	Lee et al., 2023 [26]	2/39 (5.1%)	0/39 (0%)	6/39 (15.4%)	0/39 (0%)	2.3 ± 1.2	1.4 ± 1.2	KT-2000
	Yang et al., 2023 [25]	1/35 (2.9%)	0/35 (0%)	3/35 (8.6%)	0/35 (0%)	4.2 ± 2.2	3.5 ± 2.8	Telos stress

ALLR, anterolateral ligament reconstruction; ACLR, anterior cruciate ligament reconstruction; AP, anterior–posterior; SD, standard deviation; HT, hamstring tendon; NR, not reported; CI, confidence interval.

**Table 4:** Patient-reported outcome measures of included studies.

Graft Used for ALLR	Study	Lysholm Score (Mean ± SD)		IKDC Score (Mean ± SD)		Tegner Activity Scale (Mean ± SD)	
		Isolated ACLR	ACLR + ALLR	Isolated ACLR	ACLR + ALLR	Isolated ACLR	ACLR + ALLR
HT Autograft	Torkaman et al., 2024 [33]	89.1 ± 8.7	90.2 ± 9.3	79 ± 9.2	81.5 ± 8.8	NR	NR
	Gonnachon et al., 2023 [27]	80.5 ± 11.7	82.05 ± 14.6	85.6 ± 12.3	85.8 ± 13.8	6.2 ± 1.3	6.6 ± 1.6
	Laboudie et al., 2022 [29]	83.3 ± 14.3	82 ± 14.4	86.4 ± 15.2	86 ± 16.8	7	7
	Hamido et al., 2021 [30]	NR	NR	94 ± 4.5	96 ± 5	7.8 ± 1.4	7.9 ± 0.8
	Goncharov et al., 2019 [31]	90.3 ± 10	96.3 ± 3.6	92.1 ± 10.5	97.4 ± 2.3	NR	NR
	Helito et al., 2018 [32]	87.1 ± 9	92.7 ± 5.9	90 ± 7.1	95.4 ± 5.3	NR	NR
Tibialis Allograft	Lee et al., 2023 [26]	82.9 ± 8.8	87.1 ± 9.8	87.3 ± 7.9	90.7 ± 8.1	5.6 ± 1.3	6.1 ± 1.4
	Yang et al., 2023 [25]	84.3 ± 15.9	84.7 ± 12.2	89.6 ± 17.6	89.7 ± 13.3	5.5 ± 2.1	6 ± 1.7

ALLR, anterolateral ligament reconstruction; ACLR, anterior cruciate ligament reconstruction; SD, standard deviation; HT, hamstring tendon; NR, not reported; IKDC, Internation Knee Documentation Committee.

## Data Availability

The data presented in this study are available upon request from the corresponding author.

## Abbreviations

The following abbreviations are used in this manuscript:

ACLR	Anterior cruciate ligament reconstruction
ALLR	Anterolateral ligament reconstruction
AP	Anterior–posterior
HT	Hamstring tendon
OR	Odds ratio
ACL	Anterior cruciate ligament
ALL	Anterolateral ligament
BPTB	Bone–patellar tendon–bone
PRISMA	Preferred Reporting Items for Systematic Reviews and Meta-analyses
IKDC	International Knee Documentation Committee
RCT	Randomized controlled trial
MINORS	Methodological Index for Non-Randomized Studies
MD	Mean difference
CI	Confidence interval

## Appendix A

**Table A1:** Risk of bias was assessed using the MINORS score.

Study	Torkaman et al. [33]	Gonnachon et al. [27]	Lee et al. [26]	Yang et al. [25]	Laboudie et al. [29]	Pioger et al. [28]	Goncharov et al. [31]	Helito et al. [32]
Year	2024	2023	2023	2023	2022	2022	2019	2018
Level of evidence	2	3	3	3	3	3	2	3
1. A stated aim of the study	2	2	2	2	2	2	2	2
2. Inclusion of consecutive patients	2	2	2	2	2	2	2	2
3. Prospective collection of data	2	2	2	2	2	2	2	2
4. Endpoint appropriate to the study aim	2	2	2	2	2	2	2	2
5. Unbiased evaluation of endpoints	2	2	2	2	2	2	2	2
6. Follow-up period appropriate to the major endpoint	2	2	2	2	2	2	2	2
7. Loss to follow up not exceeding 5%	2	2	0	0	1	2	2	0
8. Prospective calculation of the sample size	2	0	0	2	1	0	0	1
9. A control group having the gold standard intervention	2	2	2	2	2	2	2	2
10. Contemporary groups	2	1	1	2	2	2	2	1
11. Baseline equivalence of groups	2	2	2	2	2	2	1	2
12. Statistical analyses adapted to the study design	2	2	2	2	2	2	2	2
Total scores	24	21	19	22	22	22	21	20

**Table A2:** Inclusion criteria of studies.

Study	Year	Graft Used for ALLR
Torkaman et al. [33]	2024	Patients had unilateral ACL injury, underwent ACLR using a quadruple-bundle semitendinosus tendon autograft, had an intact meniscus, exhibited a pivot shift test grade of 2+ or 3+, and had a minimum follow-up of two years.
Gonnachon et al. [27]	2023	Patients had ACL rupture and participated in pivot contact sports such as team sports or martial arts, regardless of competition level.
Lee et al. [26]	2023	Female patients had ACL rupture and a high-grade pivot shift (grade 2) and underwent combined ACLR and ALLR.
Yang et al. [25]	2023	Patients undergoing primary ACLR + ALLR met one or more of the following criteria: participation in high-level sporting activities (training at least twice a week, competing to win, professional or elite players), involvement in pivoting sports, presence of ligamentous hyperlaxity/genu recurvatum based on the modified Beighton scale, or a grade 3 pivot shift.
Laboudie et al. [29]	2022	Patients had a pivot shift grade ≥2, hyperlaxity (knee recurvatum >10°), were high-level athletes, or had a Segond fracture.
Pioger et al. [28]	2022	Patients underwent combined ACLR + ALLR due to high-grade pivot shift, chronic injuries, hyperlaxity, or participation in pivoting sports at a young age.
Hamido et al. [30]	2021	Patients had a high-grade pivot shift (grade III), a Segond fracture, participated in high-level sports, or engaged in sports involving frequent pivoting.
Goncharov et al. [31]	2019	Patients trained at least three times a week, participated in competitions, were involved in professional sports, and were aged between 16 and 40 years.
Helito et al. [32]	2018	Patients had an ACL injury for more than 12 months, confirmed by clinical and imaging examinations, and had no associated peripheral ligament injuries apart from the anterolateral corner.

The inclusion criteria presented in each study were summarized, and cases where specific criteria for combined ACLR and ALLR were provided were separately indicated. ACL, anterior cruciate ligament; ACLR, anterior cruciate ligament reconstruction; ALLR, anterolateral ligament reconstruction.
